# Weak localization and small anomalous Hall conductivity in ferromagnetic Weyl semimetal Co_2_TiGe

**DOI:** 10.1038/s41598-019-39037-0

**Published:** 2019-03-04

**Authors:** Rajendra P. Dulal, Bishnu R. Dahal, Andrew Forbes, Niraj Bhattarai, Ian L. Pegg, John Philip

**Affiliations:** 1 Department of Physics, The Catholic University of America, Washington, DC 20064 USA; 2 The Vitreous State Laboratory, The Catholic University of America, Washington, DC 20064 USA; 3 Department of Physics, South Dakota State University, Brookings, SD 57007 USA

## Abstract

Several cobalt-based Heusler alloys have been predicted to exhibit Weyl Semimetal behavior due to time reversal symmetry breaking. Co_2_TiGe is one of the predicted ferromagnetic Weyl semimetals. In this work, we report weak localization and small anomalous Hall conductivity in half-metallic Co_2_TiGe thin films grown by molecular beam epitaxy. The longitudinal resistivity shows semimetallic behavior. Elaborate analysis of longitudinal magnetoconductance shows the presence of a weak localization quantum correction present even up to room temperature and reduction in dephasing length at lower temperature. Negative longitudinal magnetoresistance is observed from 5 to 300 K, but at 300 K magnetoresistance becomes positive above 0.5 T magnetic field. The anomalous Hall effect has been investigated in these thin films. The measured anomalous Hall conductivity decreases with increasing temperature, and a small anomalous Hall conductivity has been measured at various temperatures which may be arising due to both intrinsic and extrinsic mechanisms.

## Introduction

Heusler compounds are an interesting class of materials with a wide variety of useful properties for technological applications^1–3^. Cobalt-based Heusler alloys, Co_2_XY (X = transition or rare earth elements and Y = Si, Al, Ge and Sn) have been the subject of extensive studies in the context of spintronics over the last decade^4–6^. Some of these alloys exhibit half-metallic behavior with 100% spin polarization at the Fermi edge, and have applications in spintronics^7–9^. The Co_2_TiGe (CTG) compound is one of most promising candidates for spin manipulation because it is a half-metallic compound with a Curie temperature higher than 300 K^10–12^. A number of structural, magnetic, and electrical transport studies have been carried out in bulk CTG^10–13^. Recently, band structure calculations revealed that CTG is a Weyl semimetal (WSM) with broken time reversal symmetry due to ferromagnetism^14,15^. Interestingly, the momentum space locations of the Weyl nodes in CTG can be controlled as a function of the magnetization direction. WSM can be realized in materials either by breaking crystal inversion symmetry, time reversal symmetry, or both^16^. In inversion symmetry breaking materials, angle resolved photo emission spectroscopy, and transport measurements have provided evidence for Weyl fermions and surface Fermi arcs that are characteristics of WSMs^17–20^. In CTG, which is a magnetic WSM, the ferromagnetic behavior produces negative magnetoresistance (MR) and anomalous Hall effect, which have been reported in the bulk^15,21–23^. So, understanding the WSM behavior in half-metals solely based on transport properties will be challenging. In addition, the calculated momentum locations of the Weyl nodes in CTG with (001) magnetization are considerably far away (>250 meV) from the Fermi energy, so significant effects of Weyl behavior in transport will be difficult to observe^15,21^. In this article, we have grown CTG films using molecular beam epitaxy (MBE). We have observed semimetallic behavior, low charge carrier density, negative MR with sharp cusps at low magnetic fields, and small anomalous Hall conductivity, which depends on the longitudinal conductivity in the temperature range 5 to 300 K. These observed properties in nanostructured thin film are completely different from the reported bulk properties.

**Figure 1:**
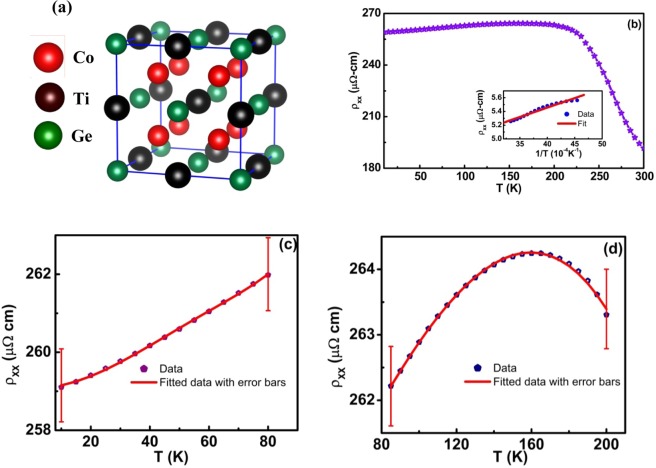
Lattice Structure and Electrical Transport. (**a**) The Fm $\bar{3}$m lattice structure of Co_2_TiGe **(b)** Resistivity variation of CTG thin film with temperature. Inset shows the resistivity variation and the fit for T > 200 K. The resistivity fitting of CTG film for temperature (**c**) less than 80 K and (**d**) 80 to 200 K.

As shown in Fig. 1(a), CTG consists of four sets of interpenetrating face-centered cubic sublattices with the space group Fm$\bar{3}$m. Smooth and continuous thin films are obtained using MBE deposition, as described in the experimental section. The typical thickness of the deposited films is 50 nm (Supplementary Information S1). The X-ray diffraction pattern recorded for CTG film is shown in Fig. S2, which can be indexed based on cubic L2_1_ structure. The detailed overview of the structural characterization of CTG is described in supplementary information. The value of the lattice constant is 5.81 Å, which matches well with previously reported values in literature^10–12^. The chemical ratio of Co:Ti:Ge is equal to 50:24.8:25.2, which is very close to the stoichiometric ratio (Fig. S3). From the magnetic measurements, saturation magnetic moment per formula unit (f.u.) calculated is 1.9 µ_B_ at 10 K, comparable to the theoretical value of 2 µ_B_/f.u^2^. At 300 K, the magnetic moment drops to 1.5 µ_B_/f.u. (Fig. S4). The magnetization versus temperature with an applied field of 500 Oe is shown in Fig. S5. A Curie temperature of 379 K is observed, while the reported values vary from 380 to 391 K^10–12^.

Figure 1(b) displays the temperature dependence of the zero-field longitudinal resistivity, ρ_xx_(T) from 10 to 300 K. Maximum resistivity is observed around 200 K. The temperature dependence of resistivity below 200 K shows a metal-like conduction^22^. For temperatures above 200 K, the resistivity is decreased. Such change of resistivity can only be found in semimetals or narrow gap semiconductors^23–26^. The logarithm of resistivity behavior above 200 K plotted against 1/T as shown in the inset can be fitted with a straight line, yielding an effective energy gap of 24 meV^27,28^. For temperatures lower than 200 K, the temperature dependence of resistivity can be written as ${\rho }_{XX}=\,{\rho }_{XX0}+{\rho }_{e-e}+{\rho }_{e-ph}+{\rho }_{e-m}$. Here, the term *ρ*_*XX*0_ is the residual resistivity which arises due to the impurity scattering; *ρ*_*e−e*_ is the resistivity contribution from electron-electron scattering, which has a T^2^ dependence at low temperature; *ρ*_*e−ph*_ term is due to electron-phonon scattering which has a T dependence at higher temperature and a T^5^ dependence at low temperature; the term *ρ*_*e−m*_ is contributed by electron-magnon (e-m) scattering. For a normal ferromagnetic material, electron-magnon scattering leads to T^2^ dependence of the resistivity^29^. However, in half-metallic ferromagnets, a band gap for minority charge carriers at Fermi level prohibits one magnon scattering. In such a case, *ρ*_*e−m*_ has a T^9/2^ dependence at low temperature and a T^7/2^ dependence at high temperature^29,30^. The T^3^ resistivity dependence has been predicted in some half-metallic systems, and such scattering process is called anomalous single magnon scattering^29^. In Co-based Heusler alloys, a T^3^ dependence has not been observed^13,29^. In our thin films, we have divided resistivity below 200 K into two regions, T < 80 K and T > 80 K. T < 80 K region is fitted with the relation *ρ*_*XX*_ = *ρ*_*XX*0_ + *AT*^2^ + *BT*^4.5^ + *CT*^5^ as displayed in Fig. 1(c). From the best fit, we have obtained *ρ*_*XX*0_ = 260 μΩ cm, A = 8.31 × 10^−10^ μΩ cm K^−2^, B = −3.24 × 10^−14^ μΩ cm K^−4.5^ and C = 2.89 × 10^−15^ μΩ cm K^−5^. The very small value of coefficient C indicates considerable phonon drag effects in our system. The small value of coefficient A indicates weak electron-electron scattering and the small negative value of coefficient B hints weak negative contribution from double magnon scattering. Figure 1(d) shows the resistivity fitting for the resistivity above 80 K and less than 200 K. We have fitted this region with the relation, *ρ*_*XX*_ = *ρ*_*XX*0_ + *DT* + *ET*^3.5^ with the values *ρ*_*XX*0_ = 260 μΩ cm, D = 5.85 × 10^−8^ μΩ cm K^−1^ and E = −5.11 × 10^−14^ μΩ cm K^−3.5^ respectively. The small value of E indicates weak negative double magnon scattering contribution in our alloy at higher temperature. Thus, the transport is mainly dominated by electron-phonon scattering and weak electron-electron and electron-magnon scatterings. The resistivity behavior observed in our CTG thin films is different than that previously reported for cobalt-based Heusler alloys. First, we have observed a semimetallic behavior with a very small energy gap below the Curie temperature. Second, we have not observed any upturn in resistivity at low temperature. Previous studies have shown a resistivity upturn in the temperature range of 5 to 50 K. It has been suggested that such a minimum should occur due to a localization effect at low temperature^29,31^.

**Figure 2:**
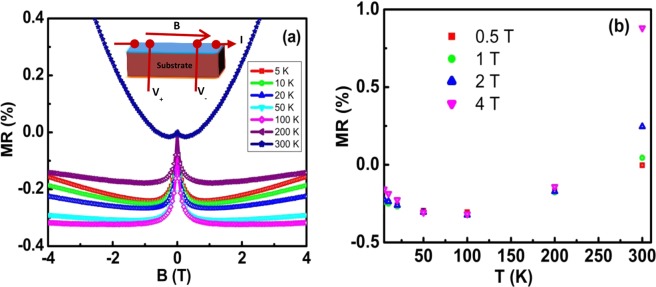
Magnetoresistance. (**a**) Magnetoresistance of CTG thin film when the magnetic field is applied along the direction of the current at different temperatures (Inset shows the measurement scheme). (**b**) MR variation of the thin film with temperatures for the four fields.

Figure 2(a) summarizes the temperature dependence of longitudinal magnetoresistance calculated using the formula $\frac{[\rho (B)-\rho (0)]}{\rho (0)}\times 100 \%$, where *ρ*(*B*) and *ρ*(0) are the resistivities at magnetic field B and zero field, respectively, and the field was applied parallel to the current as shown in the inset. The magnitude of MR is less than 0.5% at a 4 T magnetic field. The MR curves display positive cusp from 5 to 300 K at low field (<0.5 T). For fields greater than 0.5 T, MR is temperature dependent and slowly varying with magnetic field. A negative MR has been measured for temperatures up to 300 K. However, at 300 K, MR tends to go positive after a field of 0.5 T. The maximum negative MR is observed around 100 K and beyond that negative MR decreases in magnitude, as shown in Fig. 2(b). Previous studies on Co_2_TiGe have not demonstrated such sharp peak-like behavior at a low magnetic field. Indeed, they have shown non-saturating negative MR with temperature^9,22,32^ due to reduction of spin flip scattering and also, reduction of electron-magnon scattering. Studies have also shown the linear magnetic field dependence of MR^33,34^. Though negative MR has been reported in bulk CTG, it is interesting that our CTG films exhibit sharp peaks in MR behavior even up to 300 K in contrast to the bulk behavior^12^. The saturation magnitude of the MR shows an increase from 5 to 100 K that then decreases as the temperature increases above 100 K.

**Figure 3:**
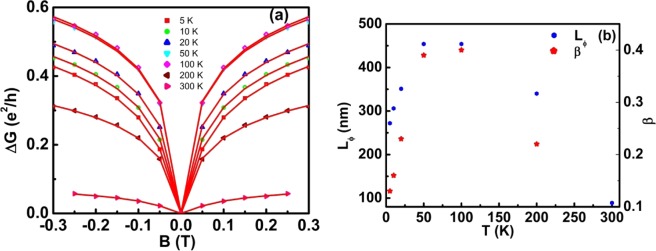
Parallel Transport. (**a**) Variation of magnetoconductance of CTG thin films at different temperatures for low fields (AA equation fitting are shown in solid red line). (**b**) Variation of dephasing length and the fitting parameter β with the temperature.

The magnetoconductance (MC) of CTG thin films is calculated as conductance at the applied field minus conductance at zero field. The increase in MC at the lower magnetic field is displayed in Fig. 3(a) at different temperatures from 5–300 K. At all temperatures, the MC curves are dominated by weak localization effects leading to a sharp downward cusp which indicates a large increase in conductance at low magnetic fields. The saturation magnitude of the conductance increases until the temperature is 100 K and then shows a reduction. Dugaev *et al*.^35^ have theoretically demonstrated that in the case of ferromagnets, spin–orbit interaction can only give negative magnetoresistance, which means MC increases with increasing magnetic field. The quantum corrections to the MC in ferromagnets when the external magnetic field applied parallel to the current can be described by the Al’tshuler and Arnov (AA) equation^36^. The conductance has a logarithmic dependence on magnetic field $\,{\rm{\Delta }}G=\frac{{e}^{2}}{2{\pi }^{2}\hslash }\,\mathrm{ln}(1+\frac{\beta e{t}^{2}{B}^{2}}{4\hslash {B}_{\varnothing }})$, where t is film thickness $\hslash =h/2\pi$, *h* is Planck’s constant, e is the electronic charge, *β* is related to the ratio of mean free path and film thickness, and *B*_∅_ is the effective dephasing field from which effective dephasing length (*L*_∅_) can be estimated as ${B}_{\varnothing }=(\hslash /4e{L}_{\varnothing }^{2})$^37^. The MC behavior at all temperatures fit well to the Al’tshuler and Arnov equation (shown in red solid line) at lower field as illustrated in Fig. 3(a). From the fits, we have calculated the dephasing length at different temperatures, which are plotted in Fig. 3(b) together with the fitting parameter β. β varies from ~0.1–0.4; these values are close to the upper bound of β in the Al’tshuler-Arnov regime, 1/3. The dephasing length shows an anomalous behavior as a function of temperature. At 5 K, the dephasing length is 270 nm. It increases with temperature, reaches a maximum value of 450 nm at 100 K, and then decreases with a further increase in temperature. This temperature dependence of dephasing length suggests the influence of other scattering mechanisms which affect the phase coherence apart from Nyquist mechanism^38^.

**Figure 4:**
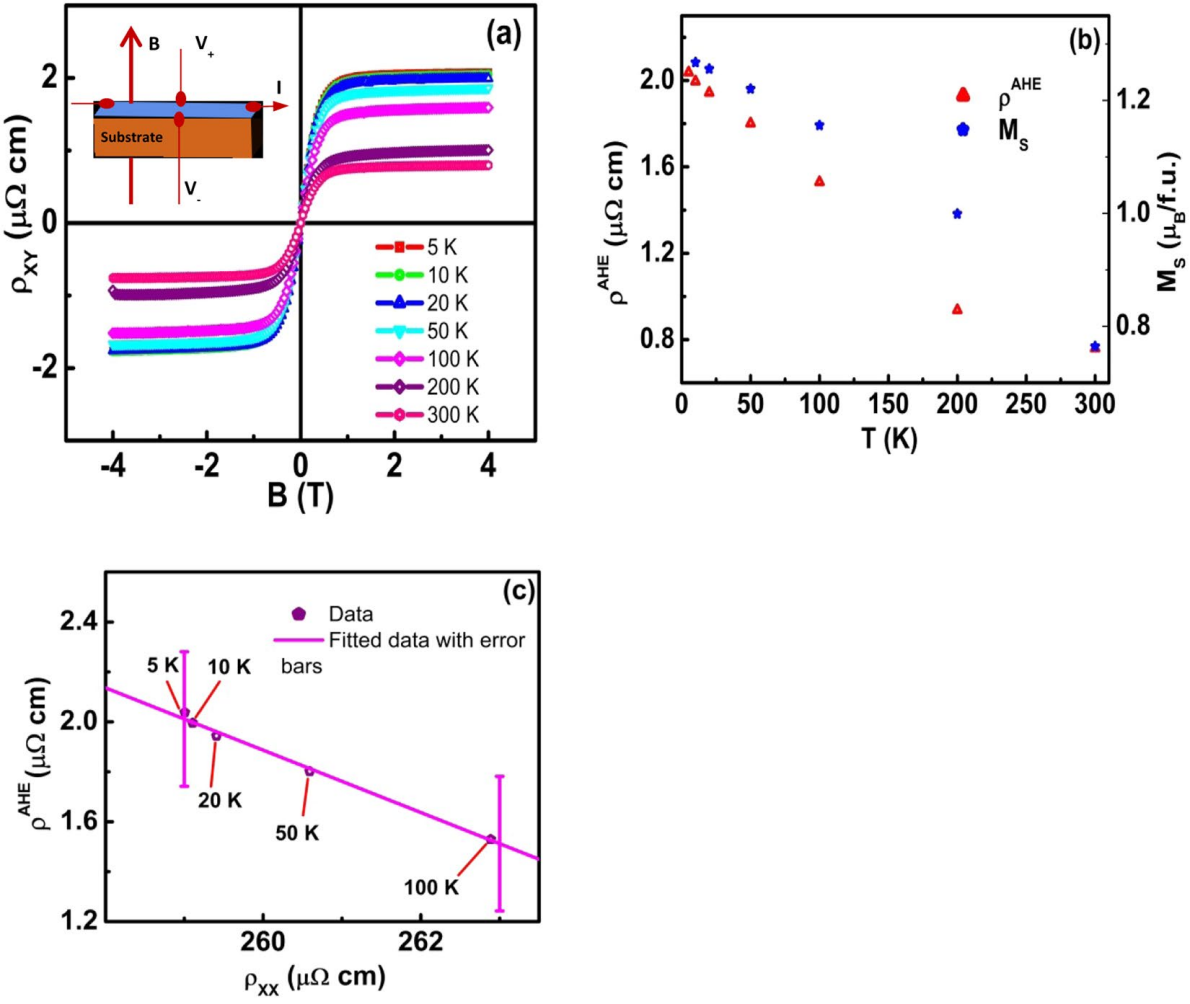
Hall Resistivity. (**a**) Hall resistivity as a function of the magnetic field of CTG thin film (Inset shows the Hall measurement scheme, where current is applied on x-direction and measured Hall voltage is perpendicular to both applied magnetic field and current). (**b**) Variation of anomalous Hall resistivity and saturation magnetization with temperature. (**c**) Plot of anomalous Hall resistivity as a function of longitudinal resistivity. The line is a fit to the data points using the equation, ${\rho }^{AHE}=(a{\rho }_{XX0}+b{\rho }_{XX0}^{2})+(a+2b{\rho }_{XX0}){\rho }_{XX(T)}+b{\rho }_{XX(T)}^{2}$.

**Figure 5:**
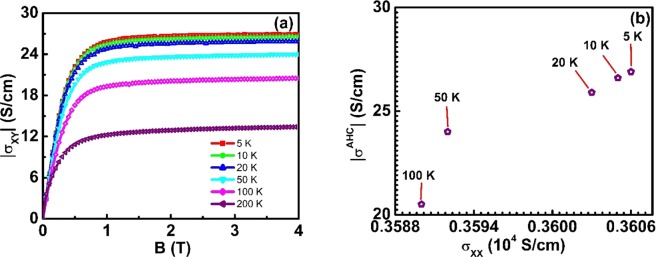
Hall Conductivity. (**a**) Variation of Hall conductivity with temperature and field. (**b**) Anomalous Hall conductivity as a function of longitudinal conductivity.

To get further insight, we have carried out Hall resistivity measurements. Figure 4(a) presents Hall resistivity (*ρ*_*XY*_) at different temperatures in the field range of ±4 T. At a particular temperature, as the applied magnetic field is increased from zero, *ρ*_*XY*_ initially increases with magnetic field before settling at saturation (Fig. S6). The total resistivity for a magnetic system can be expressed as, ${\rho }_{XY}={R}_{0}B+{\mu }_{o}{R}_{s}M$ where R_0_ and R_S_ are ordinary and anomalous Hall coefficients, M is the magnetization, and *μ*_*o*_ is the relative permeability^29,39^. The first term is the ordinary Hall resistivity that arises due to Lorentz force, and the second term is anomalous Hall resistivity (*ρ*^*AHE*^) which originates from the intrinsic magnetization. From the theoretical perspective, *ρ*^*AHE*^consists of both an intrinsic band structure dependent contribution and an extrinsic skew and side jump scattering contribution. The anomalous Hall resistivity can be extracted by a zero-field extrapolation of high field *ρ*_*XY*_ data. At a given temperature, the slope of the high field linear part of the *ρ*_*XY*_ plot is equal to R_0_. We have obtained positive values of R_0_ which increase with temperature. Based on the one band model, we have calculated the effective charge carrier density using the formula n = 1/(R_0_ e)^29,40^. The corresponding effective carrier concentration is 6.86 × 10^21^ per cm^3^ at 5 K. The mobility is another important quantity for the charge transport which can be expressed as $\mu =\frac{{R}_{o}}{{\rho }_{xx}}$. The value of mobility is 35 cm^2^V^−1^s^−1^ at 5 K. The temperature dependence of anomalous Hall resistivity and saturation magnetization is displayed in Fig. 4(b). The value of *ρ*^*AHE*^ increases as we decrease the temperature from 300 to 5 K, similar to the temperature dependence of magnetization, which clearly shows the dependence of anomalous Hall resistivity on the magnetization. To gain more understanding about the dependence of anomalous Hall resistivity on different scattering mechanisms, we have investigated how *ρ*^*AHE*^ scales with longitudinal resistivity. The scaling relation of *ρ*^*AHE*^ with *ρ*_*xx*_ for Heusler alloys has been analyzed by Vidal *et al*.^39^. The scaling relation can be written as, ${\rho }^{AHE}=(a{\rho }_{XX0}+b{\rho }_{XX0}^{2})+(a+2b{\rho }_{XX0}){\rho }_{XX(T)}+b{\rho }_{XX(T)}^{2}$, where *ρ*_*XX*0_ is the residual longitudinal resistivity and *ρ*_*XX*(*T*)_ is temperature dependent resistivity, *a* is the parameter which contains information about skew-scattering and *b* is related to intrinsic mechanism which contains also side jump contribution. The fitting is displayed in Fig. 4(c). From the fitting, we have obtained the value of *a* = 0.12 and *b* = −5 × 10^−4^ (μΩ cm)^−1^ respectively. Such negative values of *b* have been reported by Vidal *et al*.^39^ and Imort *et al*.^41^ in cobalt-based Heusler alloys because of negative scattering potential. In our thin film, though the value of *b* is smaller than the value of *a*, the fit illustrated that it could not be negligible. That means *b* which has information about side jump scattering and intrinsic mechanism contribute to anomalous Hall resistivity along with skew scattering. Hence, anomalous Hall resistivity in our thin film is contributed by extrinsic and intrinsic scattering mechanisms. The Hall conductivity is calculated using the formula, ${\sigma }_{XY}=-\,\frac{{\rho }_{XY}}{{\rho }_{XX}^{2}}$, where *ρ*_*XX*_ is longitudinal resistivity^42^ as displayed in Fig. 1(b). The field dependence of Hall conductivity is shown in Fig. 5(a). The anomalous Hall conductivity (AHC) can be extracted from this plot by extrapolating the high field Hall conductivity to zero field. We have obtained a value of 27 S/cm at 5 K. The dependence of AHC (*σ*^*AHC*^) on longitudinal conductivity (*σ*_*xx*_) is illustrated in Fig. 5(b) which demonstrates a nonlinear dependence of *σ*^*AHC*^ on *σ*_*xx*_. Further, the anomalous Hall conductivity decreases while increasing the temperature similar to the behavior of magnetization with the temperature (Fig. 4(b)), which clearly demonstrates the dependence of magnetization on *σ*^*AHC*^ in our thin film. However, previous studies have shown that intrinsic anomalous Hall effect has either a linear or a non-linear dependence on magnetization^43,44^. The longitudinal conductivity in our film is in the range observed in the dirty regime of other ferromagnets^45,46^, but the scaling relation, *σ*^*AHC *^∝ *σ*_*XX*_ does not fit to our data. From Fig. 4(c), it is evident that the anomalous Hall resistivity scales with longitudinal resistivity. Such scaling of *ρ*^*AHE*^ is neither linear nor quadratic on *ρ*_*XX*_ but combination of both linear and quadratic in *ρ*_*XX*_. Further, AHC strongly depend on *σ*_*XX*_ as the temperature increases. Hence, anomalous Hall resistivity and the small value of AHC observed in our thin film is contributed by the skew-scattering, side-jump scattering, and intrinsic mechanism^47,48^.

In summary, we have systematically investigated the structural, magnetic, electrical and magnetotransport properties of Co_2_TiGe thin films. We have demonstrated the ferromagnetic and semimetallic nature of CoTiGe thin films. The detailed analyses of parallel magnetoresistance at lower fields establishes the localization correction. The shorter dephasing length has been obtained at lower temperature. The dependence of anomalous Hall conductivity on longitudinal conductivity indicates the contributions to the AHC may arise due to both intrinsic and extrinsic mechanisms.

## Sample Preparation

Co_2_TiGe (CTG) thin films were grown using molecular beam epitaxy deposition. The base pressure inside the chamber was below 9 × 10^−10^ Torr and less than 5 × 10^−9^ Torr during the film deposition^49,50^. Polished silicon (100) substrates were used for the film deposition. Prior to deposition, the silicon substrate was cleaned with distilled water, isopropyl alcohol, and acetone. After cleaning, the substrate was etched in 2% hydrofluoric acid to remove oxide from the surface. Wafers were placed inside high vacuum and preheated at 473 K for 20 minutes. A 5 nm buffer layer of magnesium oxide was deposited. A stoichiometric ratio of Co, Ti, and Ge was simultaneously deposited; with film composition and uniformity controlled by a quartz crystal rate monitor and a low deposition rate of 0.3–0.5 Å/s. The temperature of the growth was maintained at 573 K. Films were grown with a thickness of 50 nm. After growth, the films were annealed *in situ* at 773 K for 2 h. Finally, 5 nm MgO was deposited as a cap layer.

## Thin Film Characterization

The morphology of the thin films was examined by scanning electron microscopy (SEM) (JEOL JSM-5910LV). The composition was determined by energy-dispersive X-ray (EDX), [JEOL JSM-5910LV] spectroscopy. The crystal structure was determined by x-ray diffraction analysis (XRD) using a Thermo/ARL X’TRA, (Cu-*K*_α_) diffractometer. The magnetic measurements were carried out using a Quantum Design vibrating sample magnetometer (VSM).

## Transport Measurements

To measure transport properties of thin films, Hall bar devices were grown using metal contact masks with dimensions 500 × 500 μm and 50 nm thick. Gold-wire electrical leads were attached to the samples using indium. The longitudinal resistivity and Hall resistivity of the samples were measured using a physical properties measurement system (PPMS) AC Transport with horizontal rotator option.

## Supplementary information

Supplementary Information

## Data Availability

The datasets generated and/or analyzed during the current study are available from the corresponding author upon reasonable request.

## References

[1] Felser C, Wollmann L, Chadov S, Fecher GH, Parkin SSP (2015). Basics and prospective of magnetic Heusler compounds. APL Mater..

[2] Wollmann L, Nayak AK, Parkin SSP, Felser C (2017). Heusler 4.0:TunableMaterials. Annal. Rev. Mater..

[3] Zeier WG (2016). Engineering half-Heusler thermoelectric materials using zintyl chemistry. Nat. Rev. Mater..

[4] Kübler J, Fecher GH, Felser C (2007). Understanding the trend in Curie temperatures of Co_2_-based Heusler compounds: Ab initio calculations. Phys. Rev. B.

[5] Graf T, Felser C, Parkin SSP (2011). Simple rules for the understanding of Heusler compounds. Prog. Solid State Chem..

[6] Bombor D (2013). Half-metallic ferromagnetism with unexpectedly small spin splitting in the Heusler compound Co_2_FeSi. Phys. Rev. Lett..

[7] Lee SC, Lee TD, Blaha P, Schwarz K (2005). Magnetic and half-metallic properties of the full-Heusler alloys Co2TiX (X = Al, Ga; Si, Ge, Sn; Sb). J. Appl. Phys..

[8] Jourdan M (2014). Direct observation of half-metallicity in the Heusler compound Co2MnSi. Nat. Commun..

[9] Galanakis I, Dederichs PH, Papanikolaou N (2002). Slater-Pauling behavior and origin of the half-metallicity of the full-Heusler alloys. Phys. Rev. B.

[10] Barth J (2011). Anomalous transport properties of the half-metallic ferromagnets Co2TiSi, Co2TiGe and Co2TiSn. Phil. Trans. R. Soc. London, Ser. A.

[11] Barth J (2010). Itinerant half-metallic ferromagnets Co_2_TiZ (Z = Si, Ge, Sn): Ab initio calculations and measurement of the electronic structure and transport properties. Phys. Rev. B.

[12] Bainsla L, Suresh KG (2016). Spin polarization studies in half-metallic Co2TiX (X = Ge and Sn) Heusler alloys. Curr. Appl. Phys..

[13] Prathiba G, Venkatesh S, Rajagopalan M, Harish Kumar N (2011). Half-metallic Co2TiGe-a theoretical and experimental investigation. J. Magn. Magn. Mater..

[14] Chang G (2016). Room-temperature magnetic topological Weyl fermion and nodal line semimetal states in half-metallic Heusler Co2TiX (X = Si, Ge, or Sn). Sci. Rep..

[15] Wang Z (2016). Time-reversal-breaking Weyl fermions in magnetic Heusler alloys. Phys. Rev. Lett..

[16] Zyuzin AA, Burkov AA (2012). Topological response in Weyl semimetals and the chiral anomaly. Phys. Rev. B.

[17] Xu S (2015). Discovery of a Weyl fermion semimetal and topological Fermi arcs. Science.

[18] Niemann AC (2017). Chiral magnetoresistance in the Weyl semimetal NbP. Sci. Rep..

[19] Lv BQ (2015). Experimental discovery of Weyl semimetal TaAs. Phys. Rev. X.

[20] Weng H (2015). Weyl semimetal phase in non-centrosymmetric transition metal monophosphides. Phys. Rev. X..

[21] Kushwaha SK (2017). Crystal growth and stoichiometry dependent properties of the ferromagnetic Weyl semimetal ZrCo_2-X_Sn. J. Phys.: Condens. Mater.

[22] Obaida M, Westerholt K, Zabel H (2011). Magnetotransport of Cu2MnAl, Co2MnGe, and Co2MnSi Heusler alloy thin films: from nanocrystalline disordered state to long-range-ordered crystalline state. Phys. Rev. B.

[23] Jamer ME, Asaf BA, Devakul T, Heiman D (2013). Magnetic and transport properties of Mn2CoAl oriented films. Appl. Phys. Lett..

[24] Gofryk K, Kaczorowski D, Plackowski T, Leithe-Japer A, Grin Y (2005). Magnetic and transport properties of the rare-earth-based Heusler phases RPdZ and RPd2Z (Z = Sb, Bi). Phys. Rev. B.

[25] Shekhar K (2015). Extremely large magnetoresistance and ultrahigh mobility in the topological Weyl semimetal candidate NbP. Nature Phys..

[26] Zhang M, Brück E, de Boer FR, Wu G (2004). Electronic structure, magnetism, and transport properties of the Heusler alloy Fe2CrAl. J. Magn. Magn. Mater..

[27] Dahal BR, Dulal RP, Pegg IL, Philip J (2017). Topological Crystalline SnTe nanoribbons. Solid State Commun..

[28] Dahal BR, Dulal RP, Pegg IL, Philip J (2016). Electrical and transport properties of cobalt telluride nanostructures. J. Vac. Sci. Technol. B.

[29] Prestigiacomo JC, Young DP, Adams PW, Stadler S (2014). Hall effect and magnetotransport properties of Co_2_MnSi_1-X_Al_X_. J. Appl. Phys..

[30] Kubo K, Ohata N (1972). A quantum theory of double exchange I. J. Phys. Soc. Jpn..

[31] Hazra BK (2017). Evidence for the absence of electron-electron coulomb interaction quantum correction to the anomalous Hall effect in Co_2_FeSi Heusler- alloy thin films. Phys. Rev. B.

[32] Meinkart M (2011). Electronic structure of fully epitaxial Co_2_TiSn thin films. Phys. Rev. B.

[33] Ouardi S, Fecher GH, Felser C, Kübler J (2013). Realization of spin gapless semiconductors: the Heusler compound Mn2CoAl. Phys. Rev. Lett..

[34] Xu GZ (2014). Magneto-transport properties of oriented Mn2CoAl films sputtered on thermally oxidized Si substrates. Appl. Phys. Lett..

[35] Dugaev VK, Bruno P, Barnas J (2001). Weak localization in ferromagnets with spin-orbit interaction. Phys. Rev. B.

[36] Altshuler BL, Arnov AG (1981). Magnetoresistance of thin films and of wires in a longitudinal magnetic field. JETP Lett..

[37] Zhao B (2016). Weak antilocalization in C_d_3A_s_2thin films. Sci. rep..

[38] Tkáč, V. *et al*. Influence of an anomalous temperature-dependence of phase coherence length on the conductivity of magnetic topological insulators. *arXiv:1806*.*10014v* (2018). 10.1103/PhysRevLett.123.036406 31386447

[39] Vidal EV, Schneider H, Jakob J (2011). Influence of disorder on anomalous Hall effect for Heusler compounds. Phys. Rev. B.

[40] Nasgaosa N, Sinova J, Onoda S, MacDonald AH, Ong NP (2010). Anomalous Hall effect. Rev. Mod. Phys..

[41] Imort I–M, Thomas P, Reiss, Thomas A (2012). Anomalous Hall effect in the Co-based Heusler compounds Co_2_FeSi and Co_2_FeAl. J. Appl. Phys..

[42] Miyasato T (2007). Crossover behavior of anomalous Hall effect and anomalous Nerst effect in itinerant ferromagnets. Phys. Rev. Lett..

[43] Mathieu R (2004). Scaling of the anomalous Hall effect in Sr_1-x_Ca_x_RuO_3_. Phys. Rev. Lett..

[44] Zeng C, Yao Y, Niu Q, Weitering HH (2006). Linear magnetization dependence of the intrinsic anomalous Hall effect. Phys. Rev. Lett..

[45] Onoda S, Sugimoto N, Nagaosa N (2006). Intrinsic versus extrinsic Anomalous Hall effect in Ferromagnets. Phys. Rev. Lett..

[46] Onoda S, Sugimoto N, Nagaosa N (2008). Quantum transport theory of anomalous electric, thermoelectric, and thermal Hall effects in ferromagnets. Phys. Rev. B.

[47] Yue D, Jin X (2017). Towards a better understanding of the Anomalous Hall effect. J. Phys. Soc. Jpn..

[48] Su G (2014). Anomalous Hall effect in amorphous Co_40_Fe_40_B_20_. Phys. Rev. B.

[49] Dulal RP, Dahal BR, Forbes A, Pegg IL, Philip J (2016). Large magnetization and high Curie temperature in disordered nanoscale Fe_2_CrAl thin films. J. Magn. Magn. Mater..

[50] Dulal RP, Dahal BR, Pegg IL, Philip J (2015). Ultrahigh vacuum deposition of higher manganese silicide Mn_4_Si_7_ thin films. J. Vac. Sci. Technol. B.

