# Supplementary material for: Weak localization and small anomalous Hall conductivity in ferromagnetic Weyl semimetal Co2TiGe
Source: Sci Rep. 2019 Mar 4;9:3342. doi: 10.1038/s41598-019-39037-0 (PMC6399263; doi:10.1038/s41598-019-39037-0)
Supplement: Supplementary file 1 — Supplementary Information [file 41598_2019_39037_MOESM1_ESM.docx]

Supplementary Materials to

Weak localization and small anomalous Hall conductivity in ferromagnetic Weyl semimetal Co_2_TiGe

Rajendra P. Dulal^1,2^, Bishnu R. Dahal^3^, Andrew Forbes^1,2^, Niraj Bhattarai^1,2^, Ian L. Pegg^1,2^, & John Philip*^1,2^

^1^Department of Physics, The Catholic University of America, Washington, D. C., 20064 ^2^The Vitreous State Laboratory, The Catholic University of America, Washington, D. C., 20064 ^3^Department of Physics, South Dakota State University, Brookings, SD, 57007

*To whom correspondence should be addressed. Email: philip@cua.edu

1. **Morphological of CTG thin film**

Figure S1 shows the SEM image of uniform and continuous CTG thin film of 50 nm.

**Figure S1. Morphology**. SEM image of 50 nm CTG thin film.


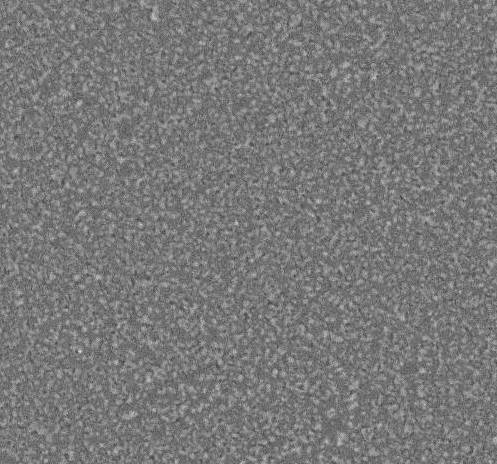


10 μm

1. **Structural characterization**


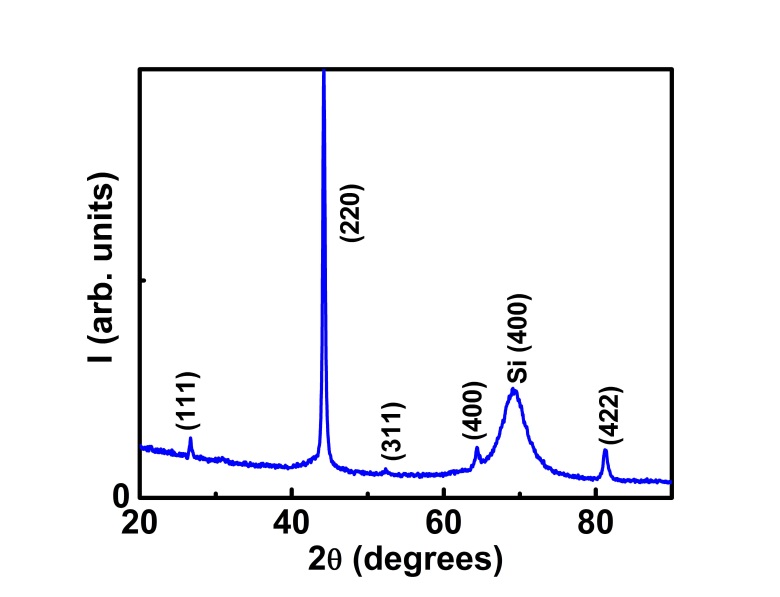


**Figure S2. Structure .** XRD pattern of CTG thin film. The broad peak at 68^o^ is from Silicon (100) substrate.

The XRD pattern is displayed in Figure S2. Co_2_XZ Heusler alloys can crystallize with different degrees of chemical order: a fully chemically ordered L2_1_ phase, a B2 phase with disorder between X and Z lattices but regular occupation on the Co atomic sites, or a fully disordered A2 phase with random occupation of the Co, X and Z atomic sites [1]. Using XRD spectrum, the ordered L2_1_ structure can be characterized by the presence of odd superlattices reflections like (111) or (311). These reflections are absent in the B2 form, which is characterized by h+k+l = 4n +2 superlattice reflections like (002). The h+k+l = 4n reflections like (004) or (422) are fundamental types and are unaffected by chemical disordering [2]. In our thin film, (111) peak is present whereas (200) peak is absent. The absence of (200) peak might indicate B32- type disorder in our thin film. However, Intensities of (200) peak could be undetectable if all the elements of the Heusler Compounds are from the same row of periodic table [2]. Hence, our alloy exhibit the L2_1_ ordered crystal structure with space group Fm$\overline{3}$m.

1. **Chemical Mapping**

Figure S3 demonstrates the chemical mapping of CTG thin film. The stoichiometric ratio of Co:Ti:Ge is equal to 50:24.8:25.2.

**Figure S3. Chemical Mapping.** Energy dispersive X-ray (EDX) spectrum of CTG film.


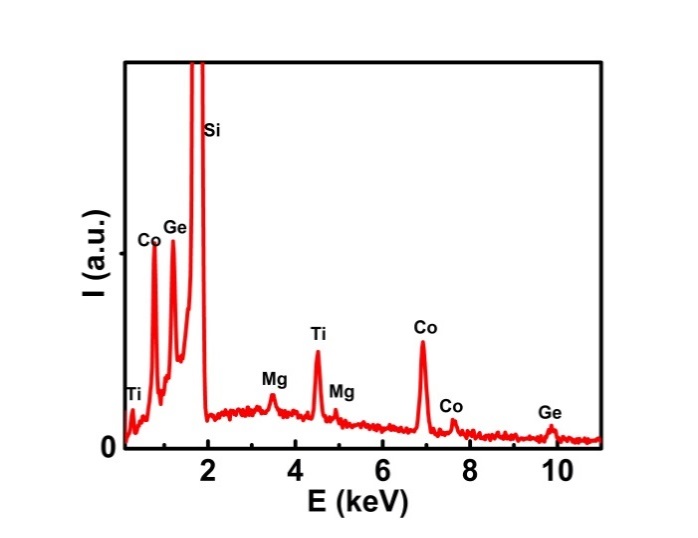


1. **Magnetic characterization – Magnetization vs Field**

**Figure S4. Magnetic Characterization.** Field variation of magnetization at 300 and 10 K


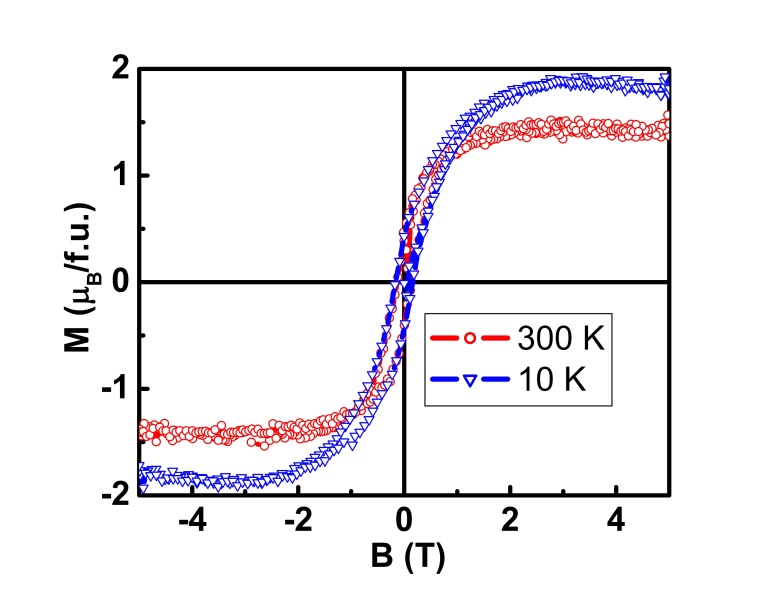


Magnetization versus magnetic field curve displays ferromagnetic behavior. The magnetic moment is reduced when the temperature is increased to 300 K.

1. **Magnetic characterization – Magnetization vs temperature**

The variation of magnetization with temperature is recorded from 10 to 400 K at a 0.05 T field. First sample is cooled to 10 K and a magnetic field of 0.05 T is applied, then the magnetization of the CTG thin film is measured up to 400 K. The transition temperature is observed at 379 K.


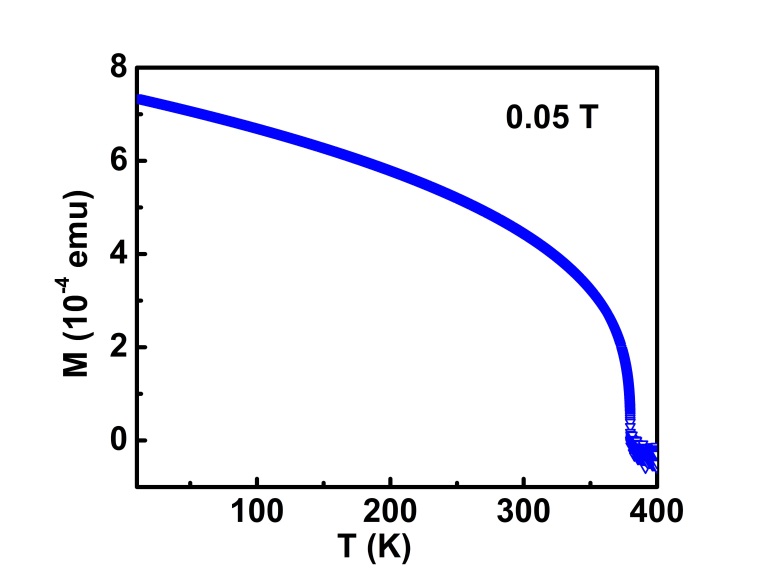


**Figure S5. Magnetic Characterization.**  Magnetization curve as a function of temperature of CTG thin film.

1. **Hall resistivity at 200 K**

**Figure S6. Hall resistivity.** Hall resistivity exhibits loop behavior.


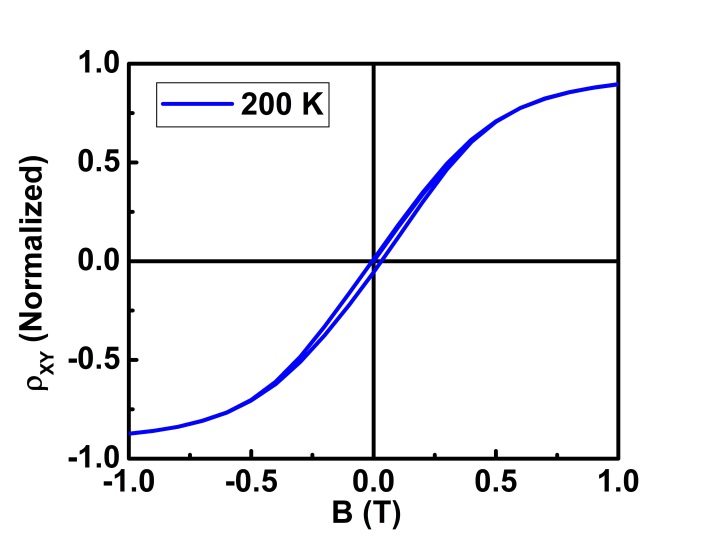


1. Webster, P., & Ziebeck, K., Magnetic Properties of Metals, Alloys and Compounds of d-elements with Main Group Elements. Part 2 in *Landolt-Bornstein- Group III Condensed Matter 19c* (ed. Wijn, H. P. J.) 75-79, doi:10.1007/b33550 (Springer, Berlin, Heidelberg 1988).
2. Graf, T., & Felser, C. Crystal Structure of Heusler Compounds in *Spintronics: From Materials to Devices* (ed. Felser, C., & Fecher, G. H.) 45-49, doi:10.1007/978-90-481-3832-63 (Springer, Netherlands, 2013
